# Survival assessment in extremely preterm neonates in a middle-income setting

**DOI:** 10.3389/fped.2025.1574613

**Published:** 2025-05-30

**Authors:** Maria J. Rodriguez-Sibaja, Olivo Herrera-Ortega, Mario I. Lumbreras-Marquez, Deneb Morales-Barquet, Sandra Acevedo-Gallegos, Yazmin Copado-Mendoza, Dulce M. Camarena-Cabrera, Juan M. Gallardo-Gaona

**Affiliations:** ^1^ Maternal-Fetal Medicine Division, Instituto Nacional de Perinatologia, Mexico City, Mexico; ^2^ Department of Epidemiology and Public Health, Universidad Panamericana School of Medicine, Mexico City, Mexico; ^3^ Neonatology Division, Instituto Nacional de Perinatologia, Mexico City, Mexico

**Keywords:** extreme prematurity, neonatal survival, viability limit, grey zone, perinatal mortality

## Abstract

**Introduction:**

Globally, an estimated 15.1 million preterm neonates are born annually, with 1% classified as extremely preterm (i.e., <28.0 weeks of gestation). The survival and outcomes of this vulnerable population are influenced by multiple factors, particularly gestational age, birth weight, and available medical resources. This study aimed to describe the hospital discharge survival of extremely preterm infants born in a middle-income setting. As a secondary objective, we assessed the neonatal morbidity associated with this group.

**Material and methods:**

In this cross-sectional study of singleton pregnancies**,** neonatal survival following extremely preterm birth was determined using three different denominators and expressed as prevalence (i.e., percentages): (1) the total number of extremely preterm births, including intrapartum fetal deaths; (2) the total number of all live births, including neonatal deaths in the delivery room, and (3) the total number of preterm neonates admitted to the neonatal intensive care unit (*N*ICU). Neonatal morbidity was assessed as a secondary outcome.

**Results:**

There were no live births between 22.0 and 23.6 weeks of gestation. Overall mortality decreased with increasing gestational age, from 100% (22/22) at <24.0 weeks of gestation to 87% (14/16), 42% (16/38), and 21% (11/52) at a gestational age of 25, 26, and 27 weeks, respectively. The survival rate to NICU discharge among extremely preterm infants was 49% (65/132), 67% (65/97), and 69% (65/93), depending on whether survival was calculated based on all births, all live births, or NICU admissions, respectively. None of the neonates born before 24.6 weeks of gestation survived to discharge. Notably, 97.0% of NICU survivors were diagnosed with major morbidity.

**Conclusion:**

The survival rate at NICU discharge exceeds 50% from 26 weeks onwards in a middle-income setting. Importantly, survival rates varied significantly depending on the denominator used, highlighting the need to carefully select inclusion criteria in neonatal survival analyses. Notably, survival after extremely preterm birth was associated with significant morbidity.

## 1 Introduction

Over the years, advances in perinatal care and management have led to sustained improvement in the survival rates and outcomes of preterm infants (1). Yet, prematurity remains one of the leading causes of neonatal mortality and long-term morbidity worldwide (2, 3). Globally, an estimated 15.1 million preterm neonates are born annually, with 1% classified as extremely preterm (i.e., <28.0 weeks of gestation) (4). Extremely preterm infants face the highest risk of mortality and morbidity, with an inverse correlation between gestational age and such risks (5). The survival and outcomes of this vulnerable population are influenced by multiple factors, particularly gestational age, birth weight, and available resources (6, 7).

The limit of viability is defined as the earliest gestational age at which a preterm neonate has a significant probability of survival with available medical technology (8). Over time, this threshold has changed, and the World Health Organization (WHO) currently defines it at 22 weeks of gestation, and/or 500 g of birth weight, and/or a birth length of 25 cm (8–10). However, this definition has inherent limitations, as the exact point at which viability occurs depends on multiple factors, including biological variability, environmental conditions, and the availability of specialized neonatal care (11, 12). These thresholds apply predominantly to high-resource settings with highly specialized technical capabilities.

Given these limitations, it is recommended that each center establishes its viability threshold to guide counseling, medical decision-making, and management of extremely preterm births. This study aimed to describe the hospital discharge survival rates of extremely preterm infants born at the *Instituto Nacional de Perinatologia*. As a secondary objective, we assessed neonatal morbidity in this population.

## 2 Material and methods

### 2.1 Study population and outcomes

This single-center cross-sectional study was conducted from January 2018 through December 2022 at the Maternal-Fetal Medicine and Neonatology Divisions of the *Instituto Nacional de Perinatologia* in Mexico City, a tertiary-level national maternal and neonatal care referral center. The study population consisted of all consecutive extremely preterm newborns (i.e., gestational age 22.0–27.6 weeks) born from a singleton pregnancy. Gestational age was primarily determined using the best obstetric estimation, defined as a reliable last menstrual period confirmed by a first-trimester crown-rump length measurement or early second-trimester biometry (i.e., biparietal diameter) (13). In cases where this information was unavailable, gestational age was estimated using standardized neonatal assessment tools (14). Births with unknown gestational age were excluded from the analysis. Stillbirths occurring outside the hospital, neonates with major structural anomalies (i.e., those expected to cause mortality or severe morbidity identifiable at birth) or genetic syndromes, cases with incomplete medical records, and neonates receiving perinatal palliative care were excluded from the study.

For the present analysis, fetal death was defined as the delivery of a neonate without signs of life (i.e., absence of breathing, heartbeat, umbilical cord pulsation, or voluntary muscle movement) after complete expulsion or extraction from the mother, occurring either before labor (i.e., antepartum mortality) or during labor and delivery (i.e., intrapartum mortality) (15). Live birth was defined as the delivery of a neonate exhibiting any sign of life following complete expulsion or extraction (15). Obstetric and neonatal management were at the discretion of the attending obstetrician and neonatologist. Notably, all live-born neonates included in this analysis received active neonatal care at birth, including cardiopulmonary resuscitation, intubation, and invasive or non-invasive mechanical ventilation (16). The Neonatal Intensive Care Unit (NICU) level at our institution is classified as level III, according to the American Academy of Pediatrics (17).

Data regarding the pregnancy, neonatal period, and infant outcomes were retrieved from electronic medical records. Definitions for neonatal morbidities are presented in Supplementary Table S1. According to institutional regulations, retrospective analyses of anonymized data are exempt from formal ethics committee review and approval. Additionally, all women provided written consent at the time of prenatal care enrollment to use their routinely collected hospital data in retrospective studies, ensuring no patient identifiers were included.

### 2.2 Statistical analysis

Baseline characteristics and morbidity are reported using *n* (%) for categorical variables, mean (standard deviation) for normally distributed continuous variables, and median (interquartile range) for non-normally distributed variables. For this descriptive analysis, the neonatal survival rate was estimated using three different denominators and expressed as percentages: (1) the total number of extremely preterm births, including intrapartum fetal deaths; (2) the total number of all live births, including neonatal deaths in the delivery room, and (3) the total number of extremely preterm neonates admitted to the NICU. A 95% confidence interval (CI) for proportions using the method proposed by Clopper-Pearson was calculated for the outcomes of interest. Statistical analyses were performed in Stata (Version 18.0, StataCorp LLC, Texas, USA).

## 3 Results

**Figure 1:**
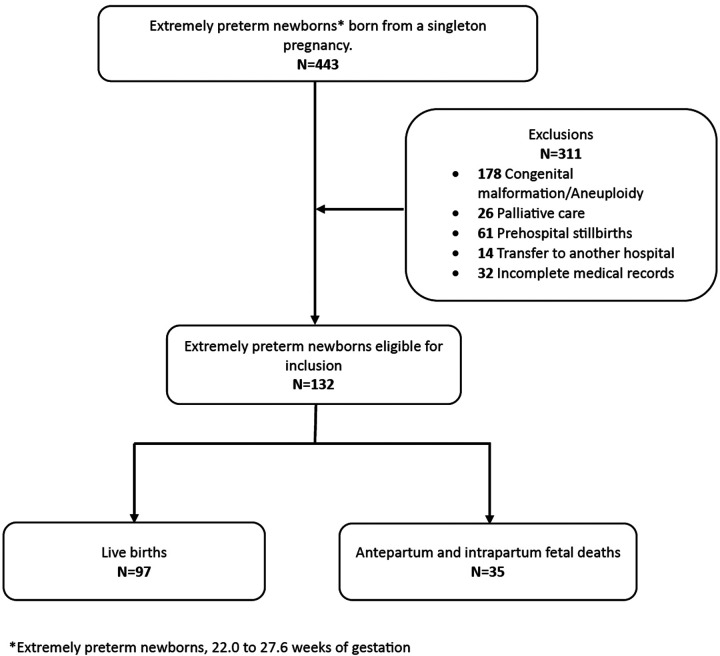
Flowchart outlining the pregnancies included in the study.

During the study period, 12,426 births were registered at the institution, with an extremely preterm birth prevalence of 3.5% (443/12,426). Data from 132 extremely preterm newborns who met the inclusion criteria were available for analysis (Figure 1). Relevant maternal demographic, obstetric, and neonatal characteristics are presented in Table 1.

**Table 1:** Demographic, obstetric, and neonatal characteristics of the study population.

Characteristic	Total cohort (*N* = 132)
Maternal age, years, median (IQR)	30 [26, 34]
Gravidity, median (IQR)	2 [1, 3]
Pregestational BMI^a^	
Underweight	4 (3.0)
Normal	57 (43.2)
Overweight	34 (25.8)
Obesity	37 (28.0)
Tobacco exposure, *n* (%)	26 (19.7)
Pregestational diabetes, *n* (%)	7 (5.3)
Gestational diabetes, *n* (%)	5 (3.8)
Chronic hypertension, *n* (%)	21 (15.9)
Hypertensive disorders of pregnancy, *n* (%)	
Gestational hypertension^b^	4 (3.0)
Preeclampsia^b^	10 (7.5)
Preeclampsia with severe features^b^	25 (18.9)
Delivery indication	
Spontaneous labor and delivery	51 (38.6)
Hypertensive disorders of pregnancy	35 (26.5)
Preterm premature rupture of membranes	30 (22.7)
Chorioamnionitis	13 (9.8)
Fetal growth restriction	9 (6.8)
Non-reassuring fetal status	1 (0.8)
Other causes	12 (9.1)
Antenatal steroids, *n* (%)	
Complete course	44 (33.3)
Incomplete course	38 (28.8)
Predelivery magnesium sulphate administration, *n* (%)^c^	88 (66.7)
Predelivery antibiotics administration, *n* (%)	72 (54.6)
Delivery mode, *n* (%)	
Spontaneous vaginal delivery	47 (35.6)
Induced labor, vaginal delivery	20 (15.2)
Cesarean delivery	65 (49.2)
Gestational age at delivery, weeks, median, (IQR)	26.3 [25.3, 27.2]
Birth weight, grams, median (IQR)	800 [660, 955]
Five-minute Apgar score, median (IQR)	7 [6, 8]
Newborn gender, *n* (%)	
Unknown	1 (0.7)
Male	76 (57.6)
Female	55 (41.7)

^a^ Calculated as weight in kilograms divided by height in meters squared.

^b^ Based on American College of Obstetricians and Gynecologists (ACOG) criteria (33).

^c^ 4 g dose bolus at least 4 h before birth.

IQR, interquartile range; BMI, body mass index.

A total of 97 (73%) live births and 35 (27%) fetal deaths were identified in the study population. Table 2 presents data on fetal mortality, live births, and mortality in the delivery room and NICU by gestational age. No live births were recorded between 22.0 and 23.6 weeks of gestation. Fetal mortality decreased from 100% (4/4) at <23.0 weeks to 4% (2/52) at 27 weeks, while live birth rates increased from 18.2% (4/22) at 24 weeks to 96% (50/52) at 27 weeks. Moreover, overall mortality declined with increasing gestational age, from 100% (22/22) at <24.0 weeks to 87% (14/16) at 25 weeks, 42% (16/38) at 26 weeks, and 21% (11/52) at 27 weeks. Notably, 27% (35/132) of overall mortality occurred before delivery, with 6% (2/35) classified as antepartum deaths and 94% (33/35) as intrapartum deaths. An additional 3% (4/132) of deaths occurred in the delivery room, while 21% (28/132) occurred in the NICU.

**Table 2:** Fetal mortality, live births, and mortality in the delivery room, and NICU by gestational age.

Gestational age (weeks)	22	23	24	25	26	27	Total
Patients, *n*	2	2	22	16	38	52	132
Fetal mortality (antepartum/intrapartum), *n* (% of all births)^a^	2 (100)	2 (100)	18 (81.8)	9 (56)	2 (5)	2 (4)	35 (27)
Live births, *n* (% of all births)	0 (0)	0 (0)	4 (18.2)	7 (44)	36 (95)	50 (96)	97 (73)
Mortality in the delivery room, *n* (% of all births)	0 (0)	0 (0)	1 (5)	1 (6)	1 (3)	1 (2)	4 (3)
Mortality in the NICU, *n* (% of all births)	0 (0)	0 (0)	3 (13)	4 (25)	13 (34)	8 (15)	28 (21)

^a^ 2 antepartum fetal deaths at 24.1 and 26.1 weeks of gestation.

NICU, neonatal intensive care unit.

### 3.1 Survival rate to NICU discharge

**Figure 2:**
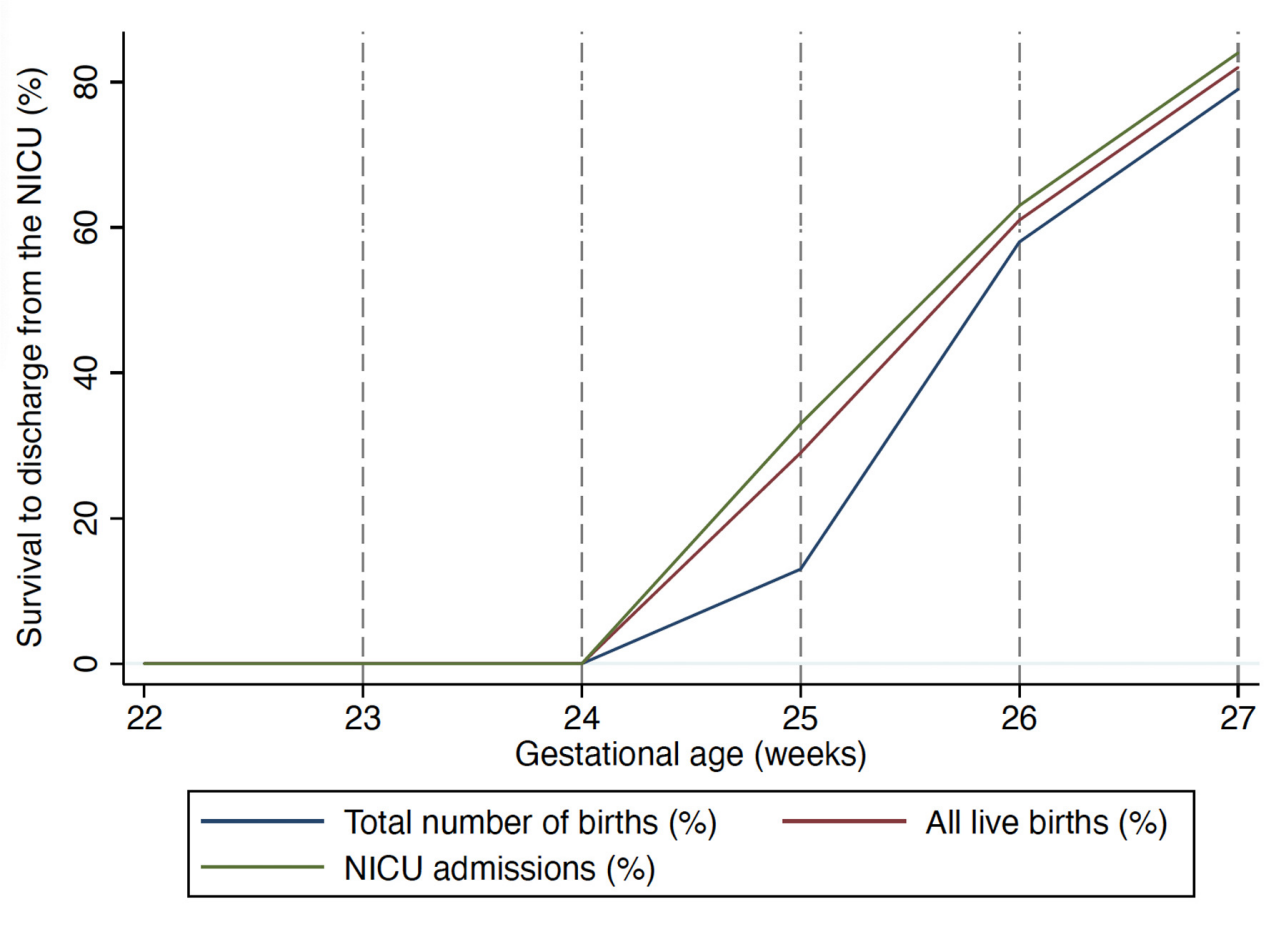
Visual representation of estimated survival rates across gestational ages for each denominator.

Table 3 presents survival rates to NICU discharge calculated using three different denominators: (1) all births (including antepartum and intrapartum fetal deaths), (2) all live births, and (3) neonates admitted to the NICU. Additionally, Figure 2 provides a visual representation of survival estimates across gestational ages for each denominator.

**Table 3:** Survival rate to discharge from the neonatal intensive care unit by gestational age.

Gestational age (weeks)	22	23	24	25	26	27	Total
(%) Total number of births, including fetal antepartum/intrapartum mortality (95% CI)	(0) 0/2	(0) 0/2	(0) 0/22	(13) 2/16 (2, 40)	(58) 22/38 (40, 70)	(79) 41/52 (70, 90)	(49) 65/132 (40, 60)
(%) All live births, including neonatal deaths in the delivery room (95% CI)	(0) 0	(0) 0	(0) 0/4	(29) 2/7 (4, 70)	(61) 22/36 (40, 80)	(82) 41/50 (70, 90)	(67) 65/97 (60, 80)
(%) Preterm newborns admitted to the neonatal intensive care unit (95% CI)	(0) 0	(0) 0	(0) 0/3	(33) 2/6 (4, 80)	(63) 22/35 (40, 80)	(84) 41/49 (70, 90)	(69) 65/93 (60, 80)

NICU, neonatal intensive care unit; CI, confidence interval (Clopper–Pearson).

The overall survival rate to NICU discharge among extremely preterm infants was 49% (65/132) when considering all births, 67% (65/97) when considering all live births, and 69% (65/93) when considering NICU admissions. No neonates born before 24.6 weeks of gestation survived to discharge. At all gestational ages, the survival rate calculated based on all births was lower than the estimated when only live births or NICU admissions were considered. However, the difference in survival rates between denominators diminished with increasing gestational age, as shown in Table 3.

### 3.2 Neonatal morbidity

Among neonates who survived NICU discharge, 96.9% (63/65) developed at least one major morbidity, defined as the presence of one or more of the following: septic shock, neonatal seizures, necrotizing enterocolitis, grade 3–4 intraventricular hemorrhage, periventricular leukomalacia, moderate-to-severe bronchopulmonary dysplasia, and retinopathy of prematurity. The most common morbidity was moderate-to-severe bronchopulmonary dysplasia, occurring in 90.8% of cases, followed by retinopathy of prematurity (75.4%) and necrotizing enterocolitis (24.6%), as shown in Table 4. Therapeutic interventions administered in the NICU are detailed in Supplementary Table S2.

**Table 4:** Major morbidity in neonates admitted to the neonatal intensive care unit.

Outcomes	Full cohort	Survivors	Non-survivors	*P*-value
	*N* = 93	*N* = 65	*N* = 28	
Gestational age at birth, weeks, median, IQR	27.0 [26.1, 27.3]	27.0 [26.4, 27.4]	26.1 [25.8, 27.0]	<0.001
Septic shock, *n* (%)	31 (33.3)	14 (21.5)	17 (60.7)	<0.001
Neonatal seizures, *n* (%)	17 (18.3)	9 (13.8)	8 (28.6)	0.090
Necrotizing enterocolitis, *n* (%)	21 (22.6)	16 (24.6)	5 (17.9)	0.470
Intraventricular hemorrhage, *n* (%)	39 (41.9)	24 (36.9%)	15 (53.6)	0.140
Grade 1–2	27 (29)	21 (32.3)	6 (21.4)	0.330
Grade 3–4	12 (12.9)	3 (4.6)	9 (32.1)	0.001
Periventricular leukomalacia, *n* (%)	12 (12.9)	8 (12.3%)	4 (14.3)	0.790
Bronchopulmonary dysplasia, *n* (%)	67 (72.0)	63 (96.9%)	4 (14.3)	<0.001
Mild	4 (4.3)	4 (6.2)	0 (0.0)	0.312
Moderate-severe	63 (67.7)	59 (90.8)	4 (14.3)	<0.001
Retinopathy of prematurity, *n* (%)	50 (53.8)	49 (75.4)	1 (3.6)	<0.001
Composite outcome^a^, *n* (%)	88 (94.6)	63 (96.9)	25 (89.2)	–^b^

^a^ The composite outcome consisted of one of the following major morbidities: septic shock, neonatal seizures, necrotizing enterocolitis, grade 3–4 intraventricular hemorrhage, periventricular leukomalacia, moderate/severe bronchopulmonary dysplasia, and retinopathy of prematurity.

^b^ The *p*-value for the composite outcome was not calculated because some neonates in the non-survivor group died before they could develop morbidities such as bronchopulmonary dysplasia or retinopathy of prematurity.

IQR, interquartile range.

## 4 Discussion

In this study, we present a comprehensive assessment of survival in extremely preterm infants born at a tertiary-level center in a middle-income setting, using different denominators for survival estimations. Our analysis showed no survival to discharge before 24.6 weeks of gestation, regardless of the denominator used. At 25 weeks, survival to discharge rates varied widely (i.e., 13%–33%) depending on the denominator. Notably, from 26 weeks onwards, survival to discharge rates exceeded 50%, and differences between denominators were no longer clinically relevant.

The findings of this study provide a detailed perspective on the reality of neonatal care for extremely preterm infants in middle-income settings. Although the observed rate of extremely preterm births was higher than national and international data (18, 19)—likely due to the tertiary-level care provided at our institution (20)—our survival results are consistent with those reported in similar settings (18, 21–23). However, they differ substantially from findings in high-income countries, where survival rates are as high as 68%, 73%, and 94% for 22, 23, and 24 weeks of gestation, respectively (24–27). These discrepancies may be explained by significant variations in active obstetric and neonatal care policies among countries, institutions, and even cultures, as well as, substantial methodological heterogeneity across studies (e.g., inclusion/exclusion criteria, definitions of live birth, etc.). While survival outcomes in high-income nations serve as a reference for improvement (28), they also underscore the significant disparities in prenatal and neonatal care resources.

### 4.1 Impact of denominator selection on survival estimations

Our analysis highlights the impact of denominator selection when reporting neonatal survival. The choice of the denominator (i.e., all births, live births, NICU admissions) considerably affects reported survival rates, particularly at lower gestational ages. At 25 weeks, survival rates varied from 13 to 33%, depending on the denominator used. However, this variation diminished with increasing gestational age, and differences became clinically irrelevant from 26 weeks onwards. A decreasing fetal mortality rate may explain this trend as gestational age increases, as well as our institution's active neonatal care policy (i.e., ≥26.0 weeks of gestation or birth weight ≥700 g). Similar findings have been reported in previous studies (1, 26, 29, 30).

The denominator used in survival analysis can significantly influence the interpretation of neonatal outcomes. Estimations based on NICU admissions may exclude neonates who did not receive resuscitation. In contrast, reports based solely on live births may overestimate survival by ignoring pregnancies at the limits of viability, where active obstetric or neonatal care is not provided (1, 29). The WHO recommends considering fetal and delivery room mortality in survival analysis, yet many studies that estimate neonatal survival fail to include this critical information (31).

### 4.2 Neonatal morbidity and healthcare implications

Neonatal morbidity at discharge was high in our study population. Moderate-to-severe bronchopulmonary dysplasia (90.8%), retinopathy of prematurity (75.4%), and necrotizing enterocolitis (24.6%) were the most frequently observed complications. This pattern has been previously described in the literature (6); however, our observed morbidity rates were significantly higher than those reported in high-income countries (24, 32). While survival rates are critical in extremely preterm neonates, long-term morbidity remains a significant concern, posing challenges for healthcare systems and families. Our findings highlight the need for improved access to high-quality neonatal care and the potential optimization of existing management strategies.

### 4.3 Strengths and limitations

Our study has some strengths. It provides updated data on a topic with limited research in middle-income settings, addressing a critical knowledge gap. We also included an unselected population with reliable gestational age assessment and all neonatal care was overseen by experienced neonatologists. Moreover, the study performed a comprehensive survival analysis using different denominators, emphasizing their impact on reported outcomes and their importance when counseling families. Furthermore, we provide precision estimates of the reported results via 95% confidence intervals.

However, some limitations should be acknowledged. First, the retrospective design may introduce biases inherent to observational studies. Second, obstetric and neonatal management were at the discretion of the attending clinicians, potentially influencing neonatal outcomes. Third, the lack of active management at periviable gestational ages may have affected survival estimates. Additionally, long-term follow-up data (e.g., neurodevelopment outcomes) were unavailable, limiting a comprehensive assessment of health trajectories beyond NICU discharge.

Finally, as this was a single-center study conducted in a national tertiary-level referral hospital, the findings reflect local clinical practices and may not be generalizable to other institutions or healthcare systems. Further studies addressing causal relationships between outcomes and exposures are warranted beyond a detailed description. Moreover, the relatively small sample size, particularly at the lowest gestational ages, hampered the possibility of additional analyses. Future studies involving larger and diverse samples are needed to validate the reported results and to account for heterogeneity in care practices across regions and countries.

## 5 Conclusions

This study demonstrated that survival rates at NICU discharge exceed 50% from 26 weeks onwards in a middle-income setting. Importantly, survival estimates vary significantly depending on the denominator used, underscoring the need for careful selection of inclusion criteria in neonatal survival analyses. Furthermore, survival following extremely preterm birth was associated with significant morbidity, reinforcing the need for ongoing efforts to optimize neonatal care.

While survival rates and outcomes for extremely preterm infants continue to improve, substantial variability in clinical outcomes and reporting methods persists across different settings. Recognizing these discrepancies highlights the importance of research that provides standardized, up-to-date data to guide neonatal care decisions.

## Data Availability

The raw data supporting the conclusions of this article will be made available by the authors, without undue reservation.
