# Supplementary material for: Survival assessment in extremely preterm neonates in a middle-income setting
Source: Front Pediatr. 2025 May 30;13:1574613. doi: 10.3389/fped.2025.1574613 (PMC12162604; doi:10.3389/fped.2025.1574613)
Supplement: Supplementary file 1 [file Table1.docx]

**Supplementary Table 1. Definitions for neonatal morbidities**

| Pathology | Definition |
| --- | --- |
| Septic shock | Cardiovascular impairment leading to a state of impaired oxygen delivery to tissues, requiring fluid resuscitation or inotropic support.[1] |
| Seizures | Sudden onset of involuntary clonic or tonic movements of one or more limbs, apnea or cyanosis, and/or lip smacking.[2] |
| Intraventricular hemorrhage | Cerebral intraventricular hemorrhage refers to subependymal germinal matrix hemorrhage, intraventricular hemorrhage, or IVH with parenchymal hemorrhage, which is classified as follows:  Grade 1 includes hemorrhage confined to the subependymal germinal matrix.  Grade 2 includes hemorrhage into the lateral ventricles without ventricular dilation.  Grade 3 involves GMH-IVH with ventricular dilation.  Grade 4 includes GMH-IVH with parenchymal involvement.[3] |
| Periventricular leukomalacia | Preterm white matter injury characterized by apparent cystic change, the histopathological substrate of which is focal macroscopic necrosis with cystic evolution. Ultrasound is very sensitive to its detection.  Diffuse periventricular leukomalacia, also known as non-cystic white matter injury, is characterized by a very diffuse inflammatory reaction. This condition is common but not accurately detected by ultrasound and is primarily diagnosed by MRI.[4,5] |
| Bronchopulmonary dysplasia | In gestational age <32 weeks, treatment with oxygen > 21% for at least 28 days plus:  Mild: Breathing room air at 36 weeks postmenstrual age or discharge, whichever comes first.  Moderate: Need for <30% oxygen at 36 weeks postmenstrual age or discharge, whichever comes first.  Severe: Need for ≥30% oxygen and/or positive pressure (positive-pressure ventilation or nasal continuous positive airway pressure) at 36 weeks postmenstrual age or discharge, whichever comes first.[6] |
| Respiratory distress syndrome | Progressive respiratory distress following birth, characterized by hypoxemia, and hypercarbia.[7] |
| Hypoglycemia | Serum glucose of 25–40 mg/dL in the first 4 hours of life.  Serum glucose of 35–45 mg/dL from 4 hours to 24 hours of life.  Serum glucose <45 mg/dL after 24 hours of life.  Serum glucose <60 mg/dL after 48 hours of life[8] |
| Hyperbilirubinemia | Elevated levels of bilirubin in the body, which, when accumulated, cause jaundice, and at excessive levels, encephalopathy. [9] |

1 Wynn JL, Wong HR. Pathophysiology and treatment of septic shock in neonates. Clin Perinatol. 2010;37(2):439–79.

2 Abend NS, Wusthoff CJ. Neonatal Seizures and Status Epilepticus. J Clin Neurophysiol. 2012;29:441–8.

3 Papile L-A, Burstein J, Burstein R, Koffler H. Incidence and evolution of subependymal and intraventricular hemorrhage: A study of infants with birth weights less than 1,500 gm. J Pediatr. 1978;92(4):529–34.

4 Agut T, Alarcon A, Cabañas F, Bartocci M, Martinez-Biarge M, Horsch S, et al. Preterm white matter injury: ultrasound diagnosis and classification. Pediatr Res. 2020 Mar;87:37–49.

5 Back SA, Miller SP. Brain injury in premature neonates: A primary cerebral dysmaturation disorder? Ann Neurol. 2014;75(4):469–86.

6 Jobe AH. Bronchopulmonary Dysplasia. 2001.Available from: www.atsjournals.org

7 Sweet DG, Carnielli VP, Greisen G, Hallman M, Klebermass-Schrehof K, Ozek E, et al. European Consensus Guidelines on the Management of Respiratory Distress Syndrome: 2022 Update. Neonatology. 2023 Mar;120(1):3–23.

8 Giouleka S, Gkiouleka M, Tsakiridis I, Daniilidou A, Mamopoulos A, Athanasiadis A, et al. Diagnosis and Management of Neonatal Hypoglycemia: A Comprehensive Review of Guidelines. Children. 2023 Jul;10(7). DOI: 10.3390/children10071220

9 National Institute for Health and Care Excellence (NICE). Jaundice in newborn babies under 28 days. 2023.Available from: www.nice.org.uk/guidance/cg98
