# Supplementary material for: Survival assessment in extremely preterm neonates in a middle-income setting
Source: Front Pediatr. 2025 May 30;13:1574613. doi: 10.3389/fped.2025.1574613 (PMC12162604; doi:10.3389/fped.2025.1574613)
Supplement: Supplementary file 2 [file Table2.docx]

**Supplementary Table 2. Management of neonates admitted to the neonatal intensive care unit (NICU)**

| Treatment | Full cohort | Survivors | Non-survivors | *P*-value |
| --- | --- | --- | --- | --- |
|  | **N = 93** | **N = 65** | **N = 28** |  |
| Surfactant administration, n (%) | 77 (82.8) | 51 (78.5) | 26 (92.9) | 0.090 |
| Mechanic ventilation, n (%) | 93 (100) | 65 (100) | 28 (100) | -- |
| Mechanic ventilation type, n (%) |  |  |  |  |
| *Noninvasive* | 6 (6.5) | 6 (9.2) | 0 (0.0) |  |
| *Invasive* | 21 (22.6) | 0 (0.0) | 21 (75.0) |  |
| *Both* | 66 (71.0) | 59 (90.8) | 7 (25.0) | <0.001 |
| Caffeine, n (%) | 87 (93.5) | 65 (100.0) | 22 (78.6) | <0.001 |
| Inotropic therapy, n (%) | 37 (39.8) | 19 (29.2) | 18 (64.3) | 0.002 |
| Postnatal steroids, n (%) | 60 (64.5) | 40 (61.5) | 20 (71.4) | 0.360 |
